# Vulnerability to acute psychosocial stress in subjects with eating disorders and history of childhood trauma: experimental evidence of a “Maltreated Ecophenotype”

**DOI:** 10.1192/j.eurpsy.2022.406

**Published:** 2022-09-01

**Authors:** E. Barone, M. Carfagno, A.M. Monteleone, V. Ruzzi, F. Pellegrino, N. Marafioti, R. Toricco

**Affiliations:** 1 University of Campania “Luigi Vanvitelli”, Department Of Psychiatry, Naples, Italy; 2 Università degli studi della Campania “L.Vanvitelli”, Psichiatria, napoli, Italy

**Keywords:** TSST, maltreated ecophenotype, ED

## Abstract

**Introduction:**

Subjects with eating disorders (ED) show a high prevalence of childhood trauma.

**Objectives:**

Aim of the study is to evaluate the emotional, biological and behavioral responses to an experimental acute psychosocial stress in subjects with ED with or without childhood maltreatment. Secondary aim is to evaluate the effects of different traumatic experiences (physical and emotional).

**Methods:**

48 women with ED completed the Childhood Trauma Questionnaire (CTQ). 29 participants (14 with Anorexia Nervosa [AN] and 15 with Bulimia Nervosa [BN]) reported an history of childhood maltreatment, while 19 (11 with AN and 8 with BN) did not. Cortisol levels, anxiety and hunger perceptions have been assessed in all participants throughout the Trier Social Stress Test (TSST) as well as body dissatisfaction after stress exposure.

**Results:**

Subjects with childhood trauma showed higher emotional reactivity and body dissatisfaction and lower hunger throughout the TSST than those without childhood trauma. Higher cortisol levels were observed in patients with AN, regardless of the presence of childhood trauma, and in those with BN and history of emotional trauma. Emotional trauma was the childhood trauma explaining most of the observed differences.

**Conclusions:**

Childhood trauma, especially emotional one, can lead to vulnerability to interpersonal stress in individuals with ED. The present study is the first that supports the “*maltreated ecophenotype”* hypothesis in subjects with ED through an experimental task and the evaluation of multiple levels of response. These data may provide new prospectives on the pathogenetic mechanisms of ED and novel therapeutic implications.

**Disclosure:**

No significant relationships.

